# Pose Estimation of a Mobile Robot Based on Fusion of IMU Data and Vision Data Using an Extended Kalman Filter

**DOI:** 10.3390/s17102164

**Published:** 2017-09-21

**Authors:** Mary B. Alatise, Gerhard P. Hancke

**Affiliations:** 1 Department of Electrical, Electronic and Computer Engineering, University of Pretoria, Pretoria 0028, South Africa; ghancke@ieee.org or gp.hancke@cityu.edu.hk; 2 Department of Computer Science, City University of Hong Kong, Hong Kong, China

**Keywords:** pose estimation, mobile robot, inertial sensors, vision, object, extended Kalman filter

## Abstract

Using a single sensor to determine the pose estimation of a device cannot give accurate results. This paper presents a fusion of an inertial sensor of six degrees of freedom (6-DoF) which comprises the 3-axis of an accelerometer and the 3-axis of a gyroscope, and a vision to determine a low-cost and accurate position for an autonomous mobile robot. For vision, a monocular vision-based object detection algorithm speeded-up robust feature (SURF) and random sample consensus (RANSAC) algorithms were integrated and used to recognize a sample object in several images taken. As against the conventional method that depend on point-tracking, RANSAC uses an iterative method to estimate the parameters of a mathematical model from a set of captured data which contains outliers. With SURF and RANSAC, improved accuracy is certain; this is because of their ability to find interest points (features) under different viewing conditions using a Hessain matrix. This approach is proposed because of its simple implementation, low cost, and improved accuracy. With an extended Kalman filter (EKF), data from inertial sensors and a camera were fused to estimate the position and orientation of the mobile robot. All these sensors were mounted on the mobile robot to obtain an accurate localization. An indoor experiment was carried out to validate and evaluate the performance. Experimental results show that the proposed method is fast in computation, reliable and robust, and can be considered for practical applications. The performance of the experiments was verified by the ground truth data and root mean square errors (RMSEs).

## 1. Introduction

Localization is identified as a problem of estimating the pose estimation (i.e., position and orientation) of a device or object such as aircraft, humans and robots, relative to a reference frame, based on sensor input. Other related problems of localization are path planning [1,2], indoor localization/navigation and tracking activities [3]. Several methods are used to determine localization: inertial sensors [4], odometry [4], GPS [4], and laser and sonar ranging sensors [5,6,7]. The use of relatively cheap sensors is important from a practical point of view; however, low-cost sensors seldom provide good performance due to measurement inaccuracies in various environments. Recently, augmented reality (AR) has been widely deployed to facilitate a new method for users to interact with their surroundings. Areas of applications of AR are tourism, education, entertainment, etc. [6,7,8,9,10]. Despite research carried out on current technologies for indoor environments to estimate the position and orientation of mobile devices, the high cost of deployment to achieve accuracy is still a major challenge. In recent times, with the Internet-of-things and mobile devices enabling sensing [11,12] for a variety of consumer, environmental and industrial applications [13,14,15,16,17], sensors and embedded intelligence have become cheaper and easier to integrate into systems [15]. The main contribution of this work is the use of SURF and RANSAC algorithms to acquire data from vision and integrate it with inertial sensors to estimate the position and orientation of the mobile robot. The inertial measurement unit (IMU) used for this practical work is the new Arduino 101 microcontroller which has both accelerometer and gyroscope compacted into the same device to give accurate results. The fusion of inertial sensors and vision-based techniques are used to provide a robust tracking experience and thus overcome the inadequacies associated with individual component-based tracking. The main advantages of this method lie in its ease of implementation, low cost, fast computation and improved accuracy. Currently, it has been proven that vision could be a promising navigation sensor that provides accurate information about position and orientation [18]. Cameras have the advantage of providing an extensive amount of information while having a low weight, limited power consumption, low cost and reasonable size. However, the use of vision methods has it shortcomings, such as illumination change and distortion due to fast movement. Inertial sensors offer good signals with high rate during fast motions but are sensitive to accumulated drift due to double integration during estimation of position. On the other hand, visual sensors provide precise ego-motion estimation with a low rate in the long term, but suffer from blurred features under fast and unpredicted motions. The aim of inertial and vision sensor integration is to overcome some fundamental limitations of vision-only tracking and IMU-only tracking using their complementary properties. Tracking of object in an environment is usually predefined with specific landmarks or markers. More discussion on markers will be presented in Section 2. The fusion methods, such as the Kalman filter or extended Kalman filter, usually adopt iterative algorithms to deal with linear and non-linear models, and hence convergence is not always assured [19,20]. For an autonomous mobile robot to localize and determine its precise orientation and position, some techniques are required to tackle this problem. Generally, the techniques are split into two categories [21,22,23,24]:

Relative localization techniques (local): Estimating the position and orientation of the robot by combining information produced by different sensors through the integration of information provided by diverse sensors, usually encoder or inertial sensors. The integration starts from the initial position and continuously update in time. The relative positioning alone can be used only for a short period of time.

Absolute localization techniques (global): This method allows the robot to search its location directly from the mobile system domain. There numerous methods usually depend on navigation beacons, active or passive landmarks, maps matching or satellite-based signals such as the global positioning system (GPS). For absolute localization, the error growth is mitigated when measurements are available. The position of the robot is externally determined and its accuracy is usually time and location-independent. In other words, integration of noisy data is not required and thus there is no accumulation of error with time or distance travelled. The limitation is that one cannot keep track of the robot for small distances (barring exceptionally accurate GPS estimates); in addition, GPS is not appropriate for indoor localization. This paper proposed to implement a hybrid method (inertial and vision) such that the weakness of one technique is complemented by the other. We conducted an indoor experiment using low-cost devices and a simple methodology to determine the pose estimation of a mobile robot in an environment in real-time. The two major components used are IMU (6-DoF) and a single camera. The system is based on the data collected from IMU and camera fused together using extended kalman filter (EKF) to determine the pose estimation of a mobile robot in reference to an object in the environment. Object identification from the image captured by the camera will be simulated and analysed using the computer toolbox in MATLAB with the speeded-up robust feature (SURF) algorithm, which is the most recent and efficient detector and descriptor for object recognition. The random sample consensus (RANSAC) algorithm will be used for the feature matching. This algorithm was used to estimate the homograph matrix of images captured. The combination of SURF and RANSAC gives robust, fast computation and accurate results for vision tracking scenarios. The accuracy of the proposed method will be shown as a result of real experiments performed and pose estimation method proposed which will be evaluated by the root mean square error model (RMSE). The RMSE shows that the pose estimation method has low error values in both position and orientation. Therefore, this approach can be implemented for practical applications used in indoor environments. In our previous work [25], we proposed a six degree of freedom pose estimation that integrates data from IMU and monocular vision. Detected natural landmarks (also known as markerless method) from image captured were used as data input for vision. Experimental results showed an improved performance of accuracy. This article is based on using a recognized object (marker-based method) captured by the camera with IMU data to determine the pose estimation of a mobile robot. The rest of the paper is organized as follows: in Section 2, a review of previous work done is presented. Section 3 discusses the proposed method for pose estimation which includes the IMU and camera mathematical expressions. The experimental setup is presented in Section 4. This is followed by Section 5, which presents the results and a discussion of the proposed method. Finally, Section 6 concludes the work and gives future directions.

## 2. Related Work

Pose estimation has been studied in past and recent times for applications in object positioning [7], robotics, and augmented reality (AR) tracking [26]. This section will discuss the existing technologies used for pose estimation in our environment these days. These methods are categorised into inertial sensor-based methods, vision sensor-based methods and fusion based methods.

### 2.1. Inertial Sensor-Based Methods

Inertial based sensor methods, also known as inertial measurement units (IMU), are comprised of sensors such as accelerometers, gyroscopes and magnetometers. Each of these sensors are deployed in robots, mobile devices and navigation systems [27,28]. The importance of using these sensors is primarily to determine the position and orientation of a particular device and/or object. An accelerometer as a sensor measures the linear acceleration, of which velocity is determined from it if integrated once; for position, integration is done twice. Results produced by an accelerometer for mobile robots have been unsuitable and of poor accuracy due to the fact that they suffer from extensive noise and accumulated drift. This can be compensated for by the use of a gyroscope. In mobile robotics, a gyroscope is used to determine the orientation by integration. The temporal gyroscope drift and bias are the main source of errors. Various data fusion techniques have been developed to overcome this unbounded error [29]. Magnetometers, accelerometers, and, more recently, vision are being used to compensate for the errors in gyroscopes. Gyroscopes as sensors measure the angular velocity, and by integrating once, the rotation angle can be calculated. Gyroscopes run at a high rate, allowing them to track fast and abrupt movements. The advantage of using gyroscope sensors is that they are not affected by illumination and visual occlusion. However, they suffer from serious drift problems caused by the accumulation of measurement errors over long periods. Therefore, the fusion of both an accelerometer and gyroscope sensor is suitable to determine the pose of an object and to make up for the weakness of one over the other.

A magnetometer is another sensor used to determine the heading angle by sensing the Earth’s magnetic field, which is combined with technologies to estimate pose estimation [30]. However, magnetometers may not be so useful for indoor positioning because of the presence of metallic objects within the environment that could influence data collected through measurements [7]. Other methods proposed to determine indoor localization include infrared, Wi-Fi, ultra-wideband (UWB), Bluetooth, WLAN, fingerprinting etc. [31]. These methods have their shortcomings; therefore, it is necessary that two or more methods should be combined to achieve an accurate result. For this work, a 6-DoF of accelerometer and gyroscope will be used as our inertial sensor to determine the pose estimation of our system. Before the IMU sensor can be used, it is necessary for the sensor device to be calibrated. The calibration procedure in ref. [32] was used along with the one given in Arduino software. This method requires the IMU board to be placed on a levelled surface to ensure stability and uprightness.

### 2.2. Vision Based Methods

Vision is another method used to determine the pose estimation of a mobile device or static objects. Vision-based methods interpret their environment with the use of a camera. The vision could be in the form of video or an image captured. This poses a spatial relationship between the 2D image captured and the 3D points in the scene. According to Genc et al. [33], the use of markers in AR is very efficient in the environment. It increases robustness and reduces computational requirements. However, there are exceptional cases where markers are placed in the area and re-calibration is needed from time to time. Therefore, the use of scene features for tracking in place of markers is reasonable especially when certain parts of the workplace do not change over time. Placing fiducial markers is a way to assist robot to navigate through its environments. In new environments, markers often need to be determined by the robot itself, through the use of sensor data collected by IMU, sonar, laser and camera. Markers’ locations are known but the robot position is unknown, and this is a challenge for tracking a mobile robot. From the sensor readings, the robot must be able to infer its most likely position in the environment. For 3D pose estimation, there are two types of methods that can be used to find the corresponding position and orientation of object or mobile robot from a 2D image in a 3D scene. They are the markerless method (also known as natural landmark) and marker-based method (also known as artificial landmark). Natural landmarks are objects or features that are part of the environment and have a function other than robot navigation. Examples are corridors, edges, doors, wall, ceiling light etc. The choice of features is vital because it will determine the complexity in the feature description, detection and matching. For the marker-based method, it requires the objects to be positioned in the environment with the purpose of robot localization. Examples of these markers can be any object but must be distinct in size, shape and colour. These makers are easier to detect and describe because the details of the objects used are known in advance. These methods are used because of their simplicity and easy setup. However, they cannot be adopted in a wide environment where large numbers of markers are deployed. For more details on vision-based tracking methods refer to [7].

#### Object Recognition and Feature Matching

Object recognition under uncontrolled, real-world conditions is of vital importance in robotics. It is an essential ability for building object-based representations of the environment and for the manipulation of objects. Object recognition in this work refers to the recognition of a specific object (e.g., a box). Different methods of scale invariant descriptors and detectors are currently being used because of their scale flexible and affine transformations to detect, recognise and classify objects. Some of these methods are oriented fast rotated BRIEF (ORB), binary robust invariant scalable keypoints (BRISK), Difference of Gaussian (DoG), fast keypoint recognition using random ferns (FERNS) [34], scale-invariant feature transform (SIFT) [35], and speeded-up robust feature (SURF) [36]. Reference [37] explains more on this. Object detection and recognition can be done through the use of computer vision, whereby an object will be detected in an image or collection of images. The recognised object is used as a reference to determine the pose of a mobile device. Basically, object detection can be categorised into three aspects: appearance-based, colour-based and feature-based. All of these methods have their advantages and limitations [38]. Here we have decided to use the feature-based technique because it finds the interest points of an object in image and matches them to the object in another image of similar scene. Generally, finding the correspondences is a difficult image processing problem where two tasks have to be solved [39]. The first task consists of detecting the points of interest or features in the image. Features are distinct elements in the images; e.g., corners, blobs, edges. The most widely used algorithm for detection includes the Harris corner detector [40]. It is based on the eigenvalues of the second moment matrix. Other types of detectors are correlation-based: the Kanade–Lucas–Tomasi tracker [41] and Laplace detector [42]. The second task is feature matching; the two most popular methods for computing the geometric transformations are the Hough transform and RANSAC algorithm [36,37,43]. RANSAC is used here because of its ability to estimate parameter with a high degree of accuracy even when a substantial number of outliers are present in the data set.

SURF was first introduced by Bay et al. [36]. SURF outperforms the formerly proposed scheme SIFT with respect to repeatability (reliability of a detector for finding the same physical interest points under different viewing conditions), distinctiveness, and robustness, yet can be computed and compared much faster. The descriptors are used to find correspondent features in the image. SURF detect interest points (such as blob) using Hessian matrix because of it high level of accuracy. This is achieved by relying on integral images for image convolutions; by building on the strengths of the leading existing detectors and descriptors (specifically, using a Hessian matrix-based measure for the detector, and a distribution-based descriptor); and by simplifying these methods to the essential. This leads to a combination of novel detection, description, and matching steps. SURF is used to detect key points and to generate its descriptors. Its feature vector is based on the Haar Wavelet response around the interested features [38]. SURF is scale-and rotation-invariant, which means that, even with variations of the size and rotation of an image, SURF can find key points.

Random sample consensus (RANSAC) is feature matcher which works well with SURF to match objects detected by SURF in images. RANSAC was first published by Fischler and Bolles [43] in 1981 which is also often used in computer vision. For example, to simultaneously unravel correspondence problems such as fundamental matrices related to a pair of cameras, homograph estimation, motion estimation and image registration [44,45,46,47,48,49]. It is an iterative method to estimate parameters of a mathematical model from a set of observed data which contains outliers. Standard RANSAC algorithm of this method is presented as follows:

Assuming a 2D image corresponds to a 3D scene points (*x_i_*, *wX_i_*), let us assume that some matches are wrong in the data. RANSAC uses the smallest set of possible correspondence and proceed iteratively to increase this set with consistent data.

- Draw a minimal number of randomly selected correspondences S_k_ (random sample);
- Compute the pose from these minimal set of point correspondences using direct linear transform (DLT);
- Determine the number C_k_ of points from the whole set of all correspondence that are consistent with the estimated parameters with a predefined tolerance. If C_k_ > C* then we retain the randomly selected set of correspondences S_k_ as the best one: S* equal S_k_ and C* equal C_k_;
- Repeat first step to third step.

The correspondences that partake of the consensus obtained from S* are the inliers. The outliers are the rest. It has to be noted that the number of iterations, which ensures a probability *p* that at least one sample with only inliers is drawn can be calculated. Let *p* be the probability that the RANSAC algorithm selects only inliers from the input data set in some iteration. The number of iterations is denoted as [50,51,52]:

$$k=\frac{\log(1-p)}{\log(1-{\left(1-w\right)}^{n})},$$

where *w* is the proportion of inliers and *n* is the size of the minimal subset from which the model parameters are estimated.

Steps to detect and recognise object (marker) in a scene are as follows:

- Load training image;
- Convert the image to grayscale;
- Remove lens distortions from images;
- Initialize match object;
- Detect feature points using SURF;
- Check the image pixels;
- Extract feature descriptor;
- Match query image with training image using RANSAC;
- If inliers > threshold then compute homograph transform box;
- Draw box on object and display.

### 2.3. Fusion of Inertial-Vision Sensor-Based Methods

The use of a single sensor is insufficient to provide accurate information of orientation or location for mobile devices, robots and objects. As each sensor has it benefits, so also they have their limitations. To complement the weakness of one sensor over another, the fusion of inertial sensors and vision is now currently being researched. Several authors have proposed different ways that the fusion of inertial sensors and vision can be integrated. The authors in [7] used only accelerometer data as the inertial sensor with vision to determine the pose estimation of an object. In [53], both the accelerometer and gyroscope data for inertial sensors were fused with a marker-based system. The use of continuously adaptive mean shift (CAMSHIFT) algorithm produces good performance but quite a lot of work has been developed using the algorithm. You et al. [54] combine the methods of fiducial and natural feature-tracking with inertial sensors to produce a hybrid tracking system of 3DoF. Data fusion was regarded as an image stabilization problem. Visual data was obtained by detecting and tracking known artificial fiducials. Visual gyro data was fused using EKF, but the work only considered the use of gyro data [55]. These proposed methods provide good performance; hence, they are used for different applications. However, combining accelerometer and gyroscope data with the recently proposed image processing algorithms SURF and RANSAC is still an open area of research. To fuse the inertial data and vision data together, several filter-based methods have been suggested in the literature. In robotic applications, pose estimation is often referred to as simultaneous localization and map building (SLAM) and has been extensively explored. SLAM has a history of adopting diverse sensor types and various motion models and a majority of the approaches have used recursive filtering techniques, such as the extended Kalman filter (EKF) [20,56], particle filter [57], unscented Kalman filter [58] and Kalman Filter. According to [20,25,27,59], EKF is the most appropriate technique to be adopted for inertial and visual fusion. Therefore, EKF is developed to fuse inertial sensor data and vision data to estimate position and orientation of a mobile robot.

## 3. Proposed Modeling Method

**Figure 1:**
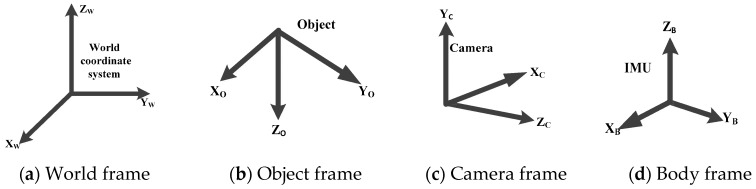
Reference coordinate system.

When working with a sensor unit containing a camera and an IMU, several reference coordinate systems have to be presented. The four major coordinate systems are depicted in Figure 1:

Reference frame for the system

- Global frame/world frame {w}: This frame aids the user to navigate and determine the pose estimation in relative to IMU and camera frames.
- IMU/body frame {b}: This frame is attached to the IMU (accelerometer and gyroscope) on the mobile robot.
- Object coordinate frame {o}: This frame is attached to the object (a 4WD mobile robot).
- Camera frame {c}: This frame is attached to the camera on the mobile robot with the x-axis pointing to the image plane in the right direction and z-axis pointing along the optical axis and origin located at the camera optical center.
- The IMU method provides orientation of the body {b} with respect to (wrt) world frame {w} *R_wb_* and vision method provides orientation of the object {o} wrt to camera frame {c} *R_co_* [26,60].

### 3.1. IMU-Based Pose Estimation

**Figure 2:**
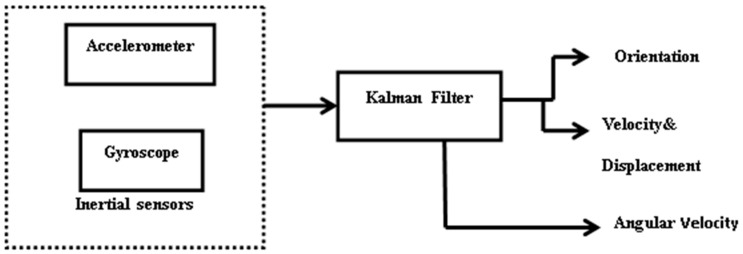
Block diagram for inertial measurement units (IMU).

Figure 2 shows the block diagram of the inertial sensors which used a Kalman filter to estimate the current pose and to reduce drifts and errors [61] of the sensors. This filter is also capable of estimating an accurate orientation of the system, but is basically used for linear systems.

Kalman filter (KF) is theoretically an ideal filter for combining noisy sensors to acquire accurate and estimated output. It is accurate because it takes known physical properties of the system into account. However, it is mathematically complex to compute and code. The calibrated accelerometer and gyroscope were used to determine orientation, angular velocity, linear velocity and displacement of the mobile robot with the use of KF. The KF was used as a prediction and correction model for the sensors.

To express an object or mobile robot orientation, several representations are proposed to be used. Examples are the axis angle, Euler angles, direct cosine matrix (DCM) and quaternions [7,26,62]. In this paper, Euler angles are adopted to solve for roll, pitch and yaw angles.

The gravity in the world frame can be obtained using coordinate information from the body frame.

(1) $$g_{w}=R_{wb}g_{b},$$

where g denotes the gravity and the subscripts b and w represents the body frame and world frame, respectively. To obtain the rotation matrix from the world frame {w} to the body frame {b}, $\left(R_{wb}\right)$, the Euler angles, roll ϕ, pitch θ, and yaw ψ can be obtained as:

(2) $$(R_{wb})=\left[\begin{array}{ccc} c\phi c\theta & -c\psi s\theta +s\psi s\phi c\theta & s\psi s\theta +c\psi s\phi c\theta \\ c\phi s\theta & c\psi c\theta +s\psi s\phi s\theta & -s\psi c\theta +c\psi s\phi s\theta \\ -s\phi & s\phi c\psi & c\phi c\theta \end{array}\right],$$

where *c* is defined as cos (), and s is defined as sin (). The world frame provides the reference frame for the body frame, in which the *x*-axis and *y*-axis are tangential to the ground and the *z*-axis is in the downward direction (view direction). The initial gravity vector in the world frame is given as:

(3) $$g_{w}=\left[\begin{array}{l} 0\\ 0\\ g \end{array}\right],$$

The 3-axis accelerometer gives the components of the gravitational accelerations expressed in the object reference frame $\left(g_{b}={\left[g_{bx}g_{by}g_{bz}\right]}^{T}\right)$, where the superscript *T* represents the transpose matrix. Hence, substituting the gravity vector is related through a rotation matrix, the relation is given as:

(4) $$g_{b}=\left[\begin{array}{l} g_{bx}\\ g_{by}\\ g_{bz} \end{array}\right]=R_{wb}g_{w}=R_{wb}\left[\begin{array}{l} 0\\ 0\\ g \end{array}\right]=\left[\begin{array}{l} -g\sin\theta \\ g\cos\varphi \sin\phi \\ g\cos\varphi \cos\theta \end{array}\right],$$

From Equation (4) pitch and roll angles can be deduced from the gravity vectors as:

(5) $$\theta =\arctan\left(\frac{g_{by}}{\sqrt{\left({g_{bx}}^{2}+{g_{bz}}^{2}\right)}}\right),$$

(6) $$\phi =\arctan\left(-\frac{g_{bx}}{g_{bz}}\right),$$

Equations to calculate position and velocity are given as:

(7) $$V_{b(k+1)}=V_{bk}+a_{bk}\Delta t,$$

(8) $$S_{b(k+1)}=S_{bk}+V_{bk}\Delta t,$$

where $a_{b}$, $V_{b}$, $S_{b}$, k,k+1 and Δt are acceleration, velocity, position, time intervals and sampling time. To calculate the angular rate we used the methods adopted in [63]. The angular rate is integrated to determine the orientation from gyroscope.

### 3.2. Vision Based Pose Estimation Method

The 3D vision-based tracking approach tracks the pose of the mobile robot with a camera in relative to the referenced object. For effective tracking, fast and reliable feature vision algorithm is vital. The process of vision localization is categorised into four major steps: acquire images via camera, detect object in the current images, match the object recognised with those contained in the database and finally, calculate the pose as a function of the recognised object. In this work, a forward-looking single camera (monocular) was used because it provides a high number of markers, thus allowing good motion estimation accuracy, if the objects are closers to the camera [19,64].

#### Projection of Object Reference Points to Image Plane

With monocular vision (one camera), a good solution in terms of scalability and accuracy is provided [65]. The monocular vision demands less calculation than stereo vision (two cameras). With the aid of other sensors such as ultrasonic sensor or barometric altimeter, the monocular vision can also provide the scale and depth information of the image frames [65,66]. The vision method provides orientation of the object {o} wrt to camera coordinate frame {c}, *R_co_* using the method in [67], to calculate the pose of the mobile robot with respect to the camera based on the pinhole camera model. The monocular vision positioning system used in [19], was used to estimate the 3D camera from the 2D image plane. The relationship between a point in the world frame and its projection in the image plane can be expressed as:

(9) λp=MP,

where λ is a scale factor, $p={\left[u,v,1\right]}^{T}$ and $P={\left[X_{w},Y_{w},Z_{w},1\right]}^{T}$ are homogenous coordinates on image plane and world coordinate M is a 3×4 projection matrix.

The above Equation (9) can further be expressed as:

(10) $$\lambda \left[\begin{array}{l} u\\ v\\ 1 \end{array}\right]=M(R_{wc}t_{wc})\left[\begin{array}{l} X_{w}\\ Y_{w}\\ Z_{w}\\ 1 \end{array}\right],$$

The projection matrix depends on the camera’s intrinsic and extrinsic parameters. The five intrinsic parameters are: focal length f, principal point $u_{0},v_{0}$ and the scaling in the image x and y directions, $a_{u}$ and $a_{v}$. $a_{u}=f_{u}$, $a_{v}=f_{v}$. The axes skew coefficient γ is often zero.

(11) $$M=\left[\begin{array}{lll} a_{u} & \gamma & u_{0}\\ 0 & a_{v} & v_{0}\\ 0 & 0 & 1 \end{array}\right],$$

Extrinsic parameters: R,T, define the position of the camera center and the camera’s heading in world coordinates. Camera calibration is to obtain the intrinsic and extrinsic parameters. Therefore, the projection matrix of a world point in the image is expressed as:

(12) $$C=-R^{-1}T=-R^{T}T,$$

where T is the position of the origin of the world coordinate, and R is the rotation matrix. For this research, camera calibration was done offline using MATLAB Calibration Toolbox [68].

### 3.3. Fusion Based on IMU and Vision Pose Estimation Method

**Figure 3:**
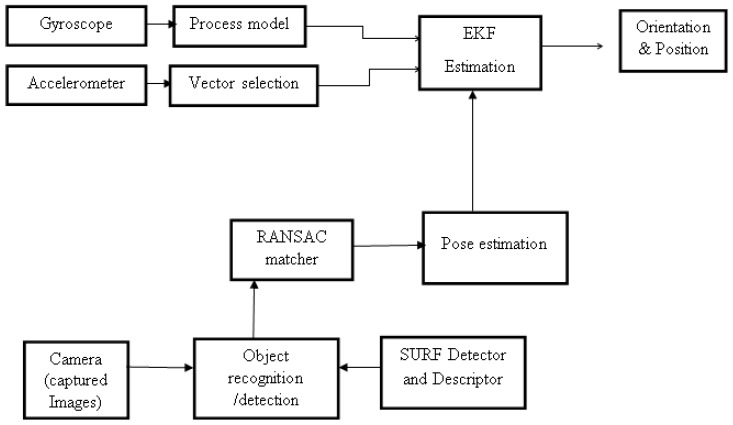
Overview of the stages of IMU and vision fusion.

The objective of sensor fusion is to improve the performance acquired by each sensor taken individually and integrating their information. Using IMU alone cannot provide accurate information, so vision is also used. The use of vision alone fails to handle occlusion, fast motion and not all areas are covered due to the field of view of the camera. Therefore, with the shortcoming of each sensor, the fusion of both IMU data and vision data will provide a better pose estimation result. Velocity, position, angular velocity and orientation are given by IMU and so also is the position and orientation given by vision. The fusion of vision and IMU is carried out using EKF. The fused EKF computed the overall pose of the mobile robot with respect to the world {w} frame. Figure 3 shows the overview stages of IMU and Vision fusion adopted.

### 3.4. EKF Implementation

For sensor fusion, EKF was implemented to estimate position and orientation from IMU and vision data. EKF is a classic approach for a nonlinear stochastic system; it uses discrete models with first-order approximation for nonlinear systems. The EKF algorithm enables complementary compensation for each sensor’s limitations, and the resulting performance of the sensor system is better than individual sensors [26,27,69]. The motion model and the observation model in EKF are established using kinematics. EKF gives reasonable performance mostly in conjunction with a long iterative tuning process. The readers can refer to [70,71] to get details of implementations and demonstrations of the EKF. The general EKF equations are given here. Let

(13) $$x_{k+1}=f_{k}({\hat{x}}_{k},\mu_{k},w_{k}),w_{k}∼N(0,Q_{k}),$$

(14) $$y_{k}=h_{k}(x_{k},v_{k}),v_{k}∼N(0,R_{k}),$$

$x_{k}$ is the state vector, $u_{k}$ denotes a known control input, $w_{k}$ denote the process noise, and $v_{k}$ is the measurement noise. $y_{k}$ is the measurement vector, *h_k_* is the observation matrix all at time *k*. The process noise $w_{k}$ has a covariance matrix Q and measurement noise $v_{k}$ has a covariance matrix *R*, are assumed to be zero-mean white Gaussian noise processes independent of each other. EKF is a special case of Kalman filter that is used for nonlinear systems. EKF is used to estimate the robot position and orientation by employing the prediction and correction of a nonlinear system model. Time prediction update equation is given as:

(15) $${{\hat{x}}_{k}}^{-}=A{\hat{x}}_{k-1}+Bu_{k},$$

(16) $$P_{k}^{-}=AP_{k-1}A^{T}+Q_{k-1},$$

where *A* is the transition matrix and *B* is the control matrix.

Measurement update equation is given as:

(17) $${{\hat{x}}_{k}}^{+}={{\hat{x}}_{k}}^{-}+K_{k}(z_{k}-H({\hat{x}}_{k}^{-})),$$

(18) $$P_{k}^{+}=(I-K_{k}H_{k})P_{k}^{-},$$

where the Kalman gain is given as:

(19) $$K_{k}=P_{k}^{-}{H^{T}}_{k}{\left(H_{k}P_{k}^{-}H_{k}+R_{k}\right)}^{-1},$$

The Jacobian matrix $H_{k}$ with partial derivatives of the measurement function *h*(·) with respect to the state *x* is evaluated at the prior state estimate ${{\hat{x}}_{k}}^{-}$, the equation is given as:

(20) $$H=\frac{\partial h}{\partial X}|X=x_{k-1},$$

For the fused filter method used in this work, we adopted one of the models used in [27]. We used accelerometer data as a control input, while gyroscope data and vision data were used as measurements. This model is extensively explained in reference above, but the process noise and covariance noise are suitably tuned. The state vector is given as:

(21) $$x={\left[p\;v\;q\;\omega \right]}^{T},$$

where p and v stand for the state variables corresponding to the 3D position and velocity of the IMU in the world frame, q denotes the orientation quaternion corresponding to the rotation matrix R and ω is the angular velocity from gyroscope. The fused transition matrix used here is given as:

(22) $$F_{1}^{1}=\left[\begin{array}{cc} F_{1} & O_{6x7}\\ O_{7x6} & F^{1} \end{array}\right],$$

The state transition matrix can be written as;

(23) $$F_{1}=\left[\begin{array}{llllll} 1 & 0 & 0 & \frac{\Delta t}{2} & 0 & 0\\ 0 & 1 & 0 & 0 & \frac{\Delta t}{2} & 0\\ 0 & 0 & 1 & 0 & 0 & \frac{\Delta t}{2}\\ 0 & 0 & 0 & 1 & 0 & 0\\ 0 & 0 & 0 & 0 & 1 & 0\\ 0 & 0 & 0 & 0 & 0 & 1\\ 0 & 0 & \frac{\Delta t}{2} & 0 & 0 & 0 \end{array}\right],$$

(24) $$F^{1}=\left[\begin{array}{lllllll} 1 & 0 & 0 & \frac{\Delta t}{2} & 0 & 0 & \frac{\Delta t^{2}}{2}\\ 0 & 1 & 0 & 0 & \frac{\Delta t}{2} & 0 & 0\\ 0 & 0 & 1 & 0 & 0 & \frac{\Delta t}{2} & \cos^{2}t\\ 0 & 0 & 0 & 1 & 0 & 0 & 0\\ 0 & 0 & 0 & 0 & 1 & 0 & \sin^{2}t\\ 0 & 0 & 0 & 0 & 0 & 1 & 0 \end{array}\right],$$

where Δt is the sampling time between images captured. $F_{1}$ and $F^{1}$ is the state transition matrix for inertial sensor and vision respectively. The process noise covariance is taken from the accelerometer and is given as:

(25) $$Q_{1}^{1}=\left[\begin{array}{ll} Q_{1} & O_{6x3}\\ O_{7x3} & Q^{1} \end{array}\right],$$

(26) $$Q_{1}=\left[\begin{array}{ll} q_{1} & O_{3x3}\\ O_{3x3} & q^{1} \end{array}\right],$$

(27) $$Q^{1}=\left[\begin{array}{lllllll} 1 & 0 & 0 & 0 & 0 & \Delta t^{2} & 0\\ 0 & 1 & 0 & 0 & 0 & 0 & \Delta t\\ 0 & 0 & 1 & 0 & 0 & 0 & 0 \end{array}\right],$$

$Q_{1}$ and $Q^{1}$ are the process noise covariance from accelerometer and vision respectively.

$q^{1}=I_{3}{\sigma_{a}}^{2}$ and 1 $q_{1}=I_{3}{\sigma_{a}}^{2}$, are the process noise taken from accelerometer while the measurement noise is taken from the gyroscope and vision where $I_{n}$ is the identity matrix dimension of *n*. R is the key matrix for sensor fusion, $R_{1}$ and $R^{1}$ are the covariance from gyroscope and vision.

$$R_{1}^{1}=\left[\begin{array}{ll} R_{1} & O_{4x3}\\ O_{3x4} & R^{1} \end{array}\right],R_{1}=I_{4}{\sigma_{g}}^{2},R^{1}=I_{3}\sigma_{v}^{2}T,$$

The observation matrix is given as;

$$H_{1}^{1}=\left[\begin{array}{l} H_{1}\\ H^{1} \end{array}\right],H_{1}=\left[O_{3x3}\;I_{3x3}\right],$$

and is the observation matrix from the gyroscope.

$$H^{1}={\left[\frac{1}{2}t^{2}R^{T}\;R\right]}^{T},$$

is the observation matrix from vision. The parameters used for filter tuning and experiments are given in Table 1.

## 4. System Hardware and Experimental Setup

**Figure 4:**
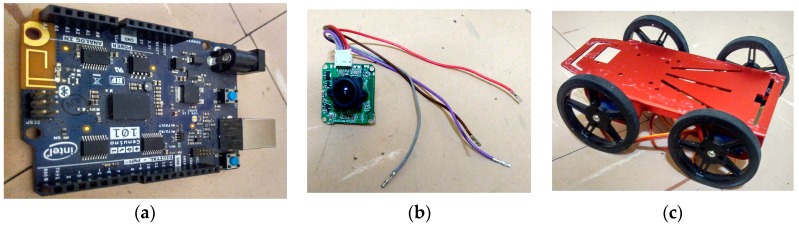
Hardware used for the experiment: (**a**) Arduino 101 microcontroller; (**b**) LinkSprite camera; (**c**) 4WD robot platform.

Figure 4 shows the major hardware used to carry out the experiment. Besides other types of components such as IR sensors, ultrasonic sensor etc. which aided robot navigation and validated the proposed method. The mobile robot used in this experiment is a four-wheel drive (4WD) as shown in Figure 4c with a working voltage of 4.8 V. Four servo motor controllers were used which allowed the robot to move up to 40 cm/s (0.4 m/s) with microcontroller (Arduino/Genuino 101) which has built-in of Inertial Measurement Unit of 3-axis accelerometer and 3-axis gyroscope, depicted in Figure 4a. As stated earlier in Section 2, the IMU was first calibrated before been coupled on the mobile robot. To reduce the payload, the frame of the robot was built with aluminium alloy. The robot was equipped with a 6 V battery to power the servo motors and a 9 V battery for the microcontroller. The mobile robot is also installed with ultrasonic sensor to measure the object distance to the mobile robot in real time. The performance issues related to reflections, occlusions, and maximum emitting angles limit independent use of ultrasonic sensors [63]. The camera was also mounted on the mobile robot to take several images from the environments. The type of camera used for this experiment is the LS-Y201-2MP LinkSprite’s new generation high-resolution serial port camera module. The pictorial representation is given in Figure 4b. Its resolution is 2 million pixels. It can capture high resolution images using the serial port. The camera is a modular design that outputs JPEG images through universal asynchronous receiver transmitter (UART), and can be easily integrated into existing design. It has a default baud rate of serial port of 115,200. More of it specification can be found in [72]. The camera was connected to the programmed microcontroller Arduino 101 mounted on the robot to capture images with a resolution of 1600 × 1200 at 6 fps. Images captured with the programme written on Arduino environment are stored in an SD card and corresponding IMU transmitted to the PC, via the USB cord which processes the images and locates the references points in the captured images. The marker (box) used as a reference object has a size of 15 × 24 cm, and was placed at a known position. The object was used to calculate the pose estimation of the mobile robot relative to the camera. The image processing and pose estimation process were analysed offline using MATLAB software. The data collected from the IMU were sent to MATLAB via the port serial. The mobile robot trajectory is designed in such a way that it moves on a flat terrain in a forward, left and right directions. The work area for the experiment is 4 m × 5.2 m.

## 5. Results and Discussion

In this section, the performance of the experiments and simulated results are evaluated and analysed. Firstly, we will present the analysis of the images captured and simulated in MATLAB. Secondly, the results of the experiments performed to determine the position and orientation of the mobile robot by fusing the inertial sensor and vision data will be presented.

### 5.1. Simulated Results of Object Detection and Recognition in an Image

In this subsection, we want to give details of the vision techniques used for detection and recognition in an image; this was implemented in MATLAB using the computer vision toolboxes following the steps given in Section 2 and with the brief introduction given in Section 4 of how images were captured and stored on an SD card and transferred to MATLAB for simulation.

**Figure 5:**
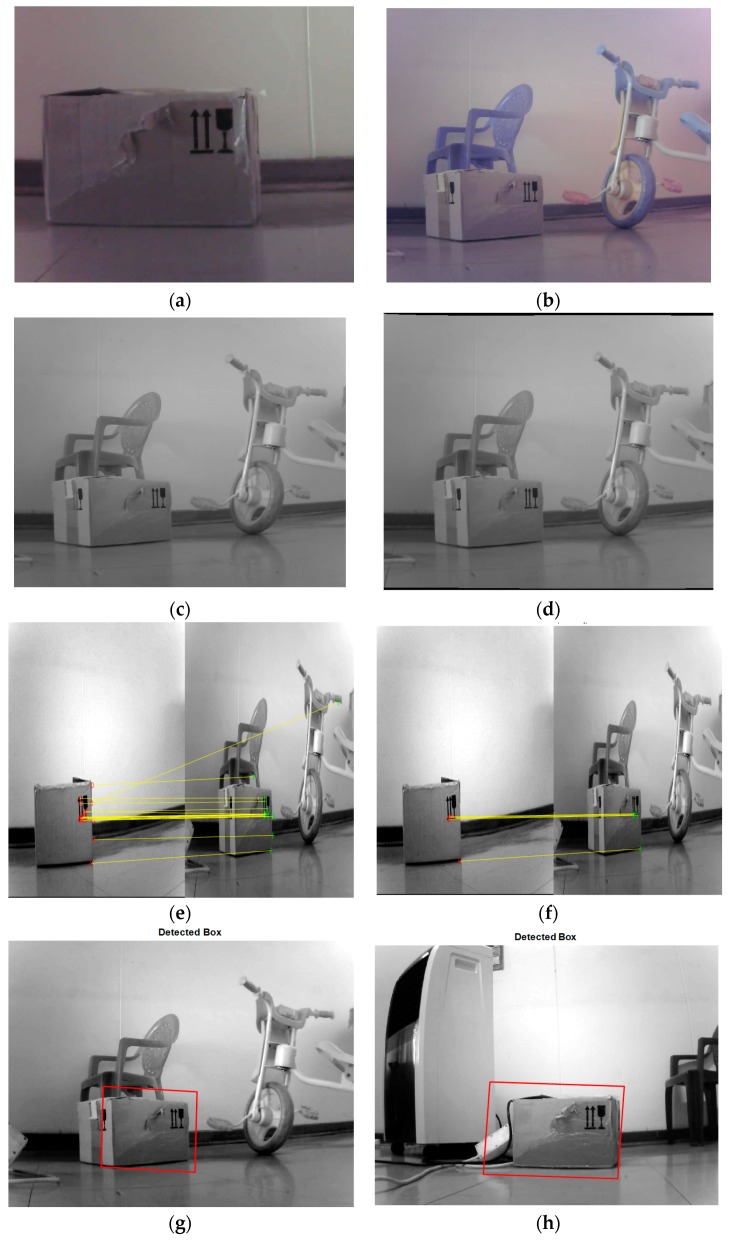
A box detected from two different images but in similar scenes. (**a**) Query image; (**b**) Training image; (**c**) Conversion of RGB to grayscale; (**d**) Removal of lens distortion; (**e**) Image including outliers; (**f**) Image with inliers only; (**g**,**h**) Images with display box around the recognised object.

More details of how the simulation was done will be given here. Figure 5 shows the detection of an object box placed in a known position to estimate the position of the mobile robot when moving in the confined area. The first step was to save the proposed object (which could also be called the query image); in this case a box was used. The image was saved in a database file. The next step was to convert the image from RGB to grayscale after resizing the image so that it would not be too large to fit on a screen.

The purpose of converting from RGB to grayscale is to acquire better results. Examples of such images are depicted in Figure 5b,c respectively. Figure 5b shows the RGB image, while Figure 5c shows the grayscale image. Some camera lenses are distorted, and therefore it is important that lens distortions are removed from images. The purpose of removing distortion in images is to correct any form of abnormalities and variations in the images to give a good quality output. Figure 5d shows an image in which distortion has been removed.

To detect features from images using SURF, Figure 5e shows a typical example of the outliers and inliers. For the simulation, 50 of the strongest feature points were extracted from the query image to match with the training image in other to have sufficient points when matching the images. The matching of images was done by RANSAC algorithm. With RANSAC algorithm, the inliers were computed in such that if the inliers points are more than the threshold then homograph transform will be estimated. This is shown in Figure 5f. The last step is for a bounding box to be designated and displayed around the recognised object as shown in Figure 5g,h.

### 5.2. Simulated Results of Object Analysis of Experimental Results

**Figure 6:**
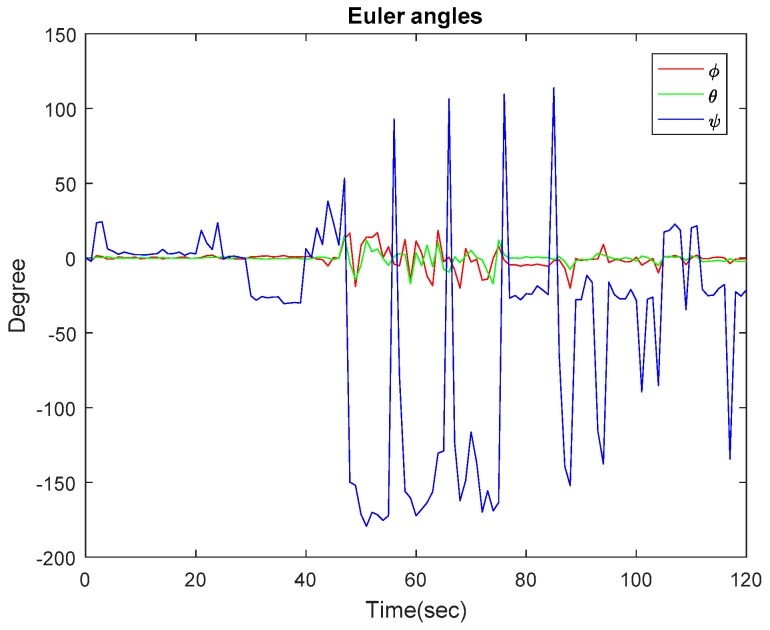
Euler angles from IMU. Roll: red; pitch: green; yaw: blue.

In this subsection, we present the results of the experiments carried out in an indoor environment. Figure 6 shows the experimental result of the Euler angles obtained from IMU and the filtered estimate. Various methods have been suggested to calculate Euler angles. Some methods considered using only data from a gyroscope to estimate Euler angles by integrating angular velocity to give orientation, while another uses only accelerometer data. Because a gyroscope measures rotation and an accelerometer does not, a gyroscope seems to be the best option to determine orientation. However, both sensors have their limitations, and therefore it is suggested that the weakness of one sensor could be complemented by the other. For this work, we combined accelerometer data and gyroscope data using a Kalman filter. Figure 2 shows the block diagram of the stages. Equations (5) and (6) were used to calculate the pitch and roll angles, while the yaw angle was calculated as an integration of angular velocity. The figure shows the robot travelling on a flat surface. It can be noted that, for about 49 s, roll and pitch angles maintained a close-to-zero angle until there was a change in direction. At the point where the robot turned 90 degrees to the right, the yaw angle was 91.25 degrees. The maximum values obtained for pitch and roll angles are 15 degrees and 18 degrees, respectively. From the experiment carried out on IMU, it can be concluded that Euler angles are a good choice for the experiment performed because the pitch angles did not attain ±90 degrees to cause what is known as Gimbal lock.

**Figure 7:**
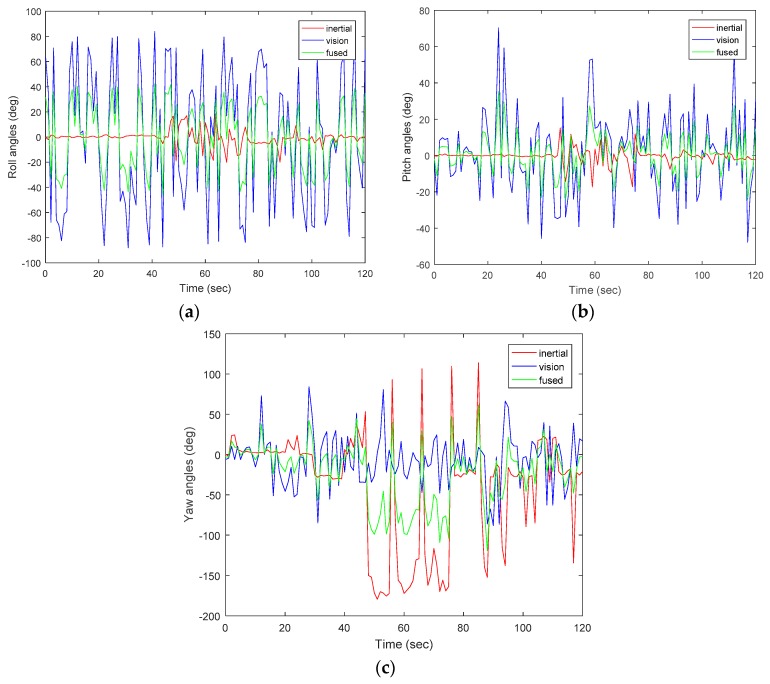
Orientation results for fused sensors. (**a**) Roll angles; (**b**) Pitch angles; (**c**) Yaw angles.

Figure 7a–c shows the orientation result of the fused data from inertial sensor and vision. The IMU was able to abruptly determine the direction of mobile, but the vision slowly captured the images to determine the orientation of the mobile robot. With different sampling frequencies, computation time did not allow both estimates to run at the same time. The IMU was able to determine the direction of the robot within a specific path, but with the camera, the rotational axis was extended to capture more views; therefore, the range of direction was widened and areas which could not be covered by IMU were captured by the camera, although vision-based tracking is more accurate for slow movement than IMU. However, using only computer vision, tracking is lost almost immediately; it is therefore obvious that the addition of IMU is beneficial. EKF is used to fuse the inertial and visual measurement to estimate the state of the mobile robot. With EKF, corrections for pose estimation were made; this shows that the filter is efficient, specifically when fusing two or more sensors together. Equations (10)–(12) from Section 3 were used to calculate the camera pose in reference to the image plane. From the equations, the intrinsic and extrinsic parameters were estimated through the camera calibration. It should be noted that the described system is very sensitive to calibration parameters. Errors in parameters used for calibration could deteriorate the tracking of the system. Hence, the design of accurate calibration methods is vital for proper operation. As observed from the figures, there is a slight difference between the data obtained from inertial sensor to that of vision. At the point where the robot made a 90 degrees right turn, the yaw value for IMU was 91.25 degrees, and 88 degrees for vision. Pitch and roll angles both have values of 1.2 degrees and 4 degrees. With the proposed method, through the use of EKF, accumulated errors and drifts were reduced and improvement was thereby achieved.

**Figure 8:**
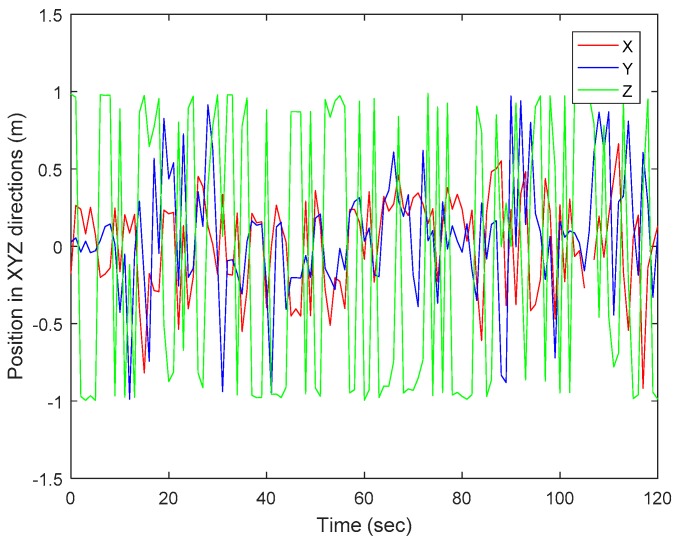
Experimental position in XYZ directions from vision data.

Figure 8 shows a comparison of the three directions of the mobile robot taken from vision only. The figure shows a distinctive estimation of position of the mobile robot. The position estimation based on the reference object in the image is relative to the position of the mobile robot and the world coordinate, with the median vector of the planar object for Z-axis close to 1 and −1. This shows that the feature selection method used is effective. Therefore, SURF and RANSAC algorithms combination can be used to determine the accurate position of an object through vision.

### 5.3. Performance: Accuracy

**Figure 9:**
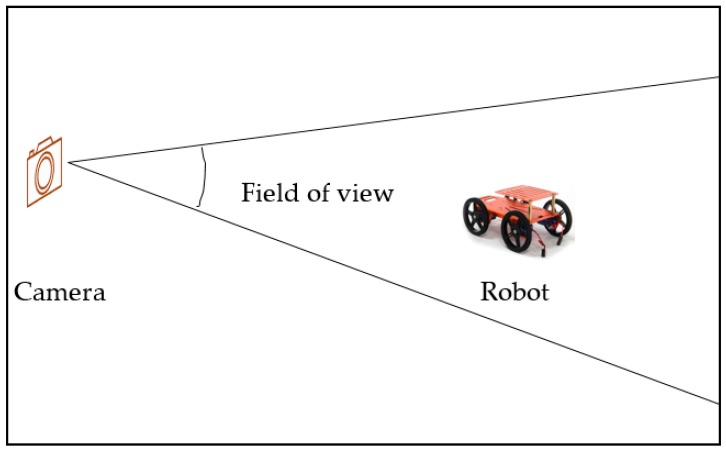
Ground truth system based on a camera.

**Figure 10:**
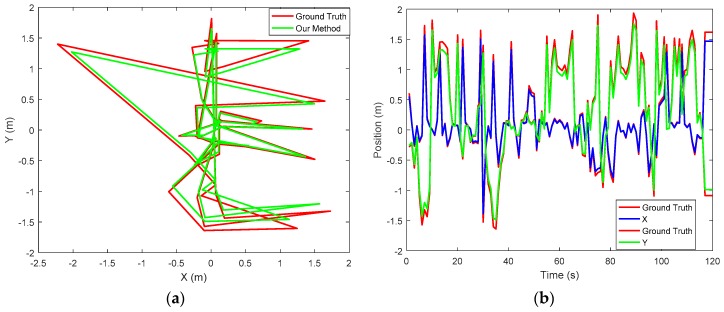
Comparing the proposed method with the ground truth. (**a**) Robot trajectory in the XY plane; (**b**) Position corresponding to the trajectory.

The ground truth data was collected with the use of external camera placed in the environment of experiment. The external camera was used because it’s less expensive, available and reliable to determine 6-DoF of position. The camera was placed on a flat terrain with the mobile robot with a distance of 4.90 m in between; the scenario is shown in Figure 9. Since the camera used was neither 360 degrees nor a motion camera, it was ensured that the camera was able to cover the experiment area. It can be observed from the figure that our method exhibits good performance, as it is close to the ground truth. However, further improvement of the proposed method is encouraged. For accurate ground truth data to be obtained, a motion capture camera or a laser ranging sensor is also suggested. The sensors are expensive, but an accurate result is guaranteed. Figure 10a shows the trajectory of the mobile robot projected in the XY plane and Figure 10b shows the corresponding positions of the mobile robot trajectory.

**Figure 11:**
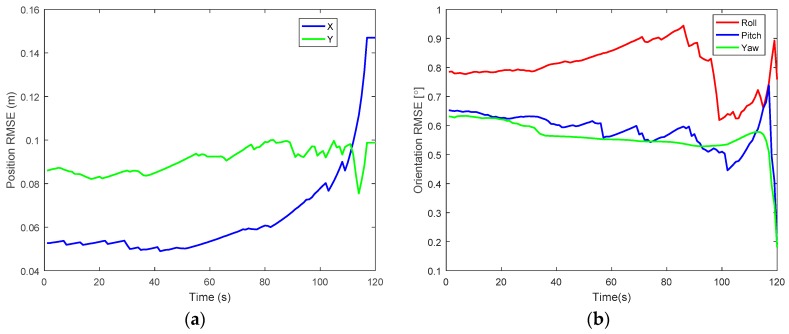
Results of RMSE for position and orientation. (**a**) Position; (**b**) Orientation.

Furthermore, the accuracy of the proposed method is assessed and evaluated by computing the root mean square error (RMSE). To evaluate the accuracy, IMU and a single camera were used as equipment for real measurement. Figure 11a,b show the results of error for position and orientation, which is the difference between the ground truth and proposed method. From the graph, it can be deduced that the maximum error value for position and orientation are 0.145 m and 0.95° respectively. These error values are still reasonable for indoor localization. In Table 2, RMSE position and orientation are further stated for specific periods. It can be observed from the table that the position error slightly increases with increase in time. For RMSE orientation, both pitch and yaw error angles decreases as time increases while for roll, error was gradually increasing from the start time to about 80 s and later decreases. The accuracy of the proposed method was improved and better performances were achieved.

## 6. Conclusions

In this paper, a novel fusion of computer vision and inertial measurements to obtain robust and accurate autonomous mobile robot pose estimation was presented for an indoor environment. The inertial sensor used is the 6-DoF, which was used to determine the linear velocity, angular velocity, position and orientation. For the computer vision, a single forward-looking camera was used to generate 2D/3D correspondences. The purpose of data fusion is to produce reliable data that is not influenced by accelerometer noise and gyroscope drift. In respect to this, vision was proposed as the best fit to complement the weaknesses of inertial sensors. The inertial sensors and the camera were both mounted on the robot to give excellent performance of the robot estimate.

For object recognition, SURF and RANSAC algorithms were used to detect and match features in images. SURF is used to detect key points and to generate its descriptors. It is scale-and rotation-invariant, which means that, even with differences on the size and on the rotation of an image, SURF can find key points. In addition, RANSAC is an algorithm to estimate the homograph matrix of an image; therefore, the combination of SURF and RANSAC gives robust, fast computation and accurate results for vision tracking scenarios.

The experimental results have shown that a hybrid approach of using inertial sensors and vision is far better than using a single sensor. An extended Kalman filter was designed to correct each sensor hitches by fusing the inertial and vision data together to obtain accurate orientation and position. RMSE values for position and orientation were determined to evaluate the accuracy of the technique. As a result, the method shows reliable performance with high accuracy. This type of system proposed further improves the accuracy with respect to localization. The weakness of this method is that it may not be a good approach to be used in a large environment, because the field of view is limited and not all areas can be covered. It is therefore important to consider the use of stereo vision (i.e., the use of two cameras). Again, another limitation is the single type of object (marker) that was used as a reference to determine the pose estimation of the mobile robot. The use of two or more mobile objects to estimate the robot’s position and orientation in other to give better and accurate results should also be considered. Further research work is to determine the robot’s pose estimation by tracking a mobile object in a real-time video in a large scale environment.

## Figures and Tables

**Table 1:** Parameters and their values for filter tuning.

Variables	Meanings
Sampling interval of IMU sensor	100 Hz
Gyroscope measurement noise variance, $\sigma_{g}$	0.001 rad^2^/s^2^
Accelerometer measurement noise variance, $\sigma_{a}$	0.001 m/s^2^
Camera measurement noise variance, $\sigma_{v}$	0.9
Sampling interval between image frames	25 Hz

**Table 2:** RMSE of position and orientation.

Time (s)	Position Error (m)		Orientation Error (Degree)		
	x	y	Roll	Pitch	Yaw
20	0.05	0.08	0.78	0.62	0.62
40	0.05	0.08	0.81	0.60	0.56
60	0.07	0.09	0.85	0.56	0.55
80	0.06	0.09	0.90	0.56	0.54
100	0.07	0.09	0.62	0.50	0.53
120	0.14	0.09	0.75	0.18	0.18
